# Exploration of Epigenetics for Improvement of Drought and Other Stress Resistance in Crops: A Review

**DOI:** 10.3390/plants10061226

**Published:** 2021-06-16

**Authors:** Chao Sun, Kazim Ali, Kan Yan, Sajid Fiaz, Richard Dormatey, Zhenzhen Bi, Jiangping Bai

**Affiliations:** 1 Gansu Provincial Key Laboratory of Aridland Crop Science, College of Agronomy, Gansu Agricultural University, Lanzhou 730070, China; sunc@gsau.edu.cn (C.S.); kazim76@gmail.com (K.A.); rmddormatey@gmail.com (R.D.); zhenzhenbigsau@gmail.com (Z.B.); 2 National Institute for Genomics and Advanced Biotechnology, National Agricultural Research Centre, Park Road, Islamabad 45500, Pakistan; 3 School of Biological and Pharmaceutical Engineering, Lanzhou Jiaotong University, Lanzhou 730070, China; yank@mail.lzjtu.cn; 4 Department of Plant Breeding and Genetics, The University of Haripur, Khyber, Pakhtunkhwa 22620, Pakistan; sfiaz@uoh.edu.pk

**Keywords:** epigenetics, drought tolerance, novel plant breeding techniques, stress tolerance improvement

## Abstract

Crop plants often have challenges of biotic and abiotic stresses, and they adapt sophisticated ways to acclimate and cope with these through the expression of specific genes. Changes in chromatin, histone, and DNA mostly serve the purpose of combating challenges and ensuring the survival of plants in stressful environments. Epigenetic changes, due to environmental stress, enable plants to remember a past stress event in order to deal with such challenges in the future. This heritable memory, called “plant stress memory”, enables plants to respond against stresses in a better and efficient way, not only for the current plant in prevailing situations but also for future generations. Development of stress resistance in plants for increasing the yield potential and stability has always been a traditional objective of breeders for crop improvement through integrated breeding approaches. The application of epigenetics for improvements in complex traits in tetraploid and some other field crops has been unclear. An improved understanding of epigenetics and stress memory applications will contribute to the development of strategies to incorporate them into breeding for complex agronomic traits. The insight in the application of novel plant breeding techniques (NPBTs) has opened a new plethora of options among plant scientists to develop germplasms for stress tolerance. This review summarizes and discusses plant stress memory at the intergenerational and transgenerational levels, mechanisms involved in stress memory, exploitation of induced and natural epigenetic changes, and genome editing technologies with their future possible applications, in the breeding of crops for abiotic stress tolerance to increase the yield for zero hunger goals achievement on a sustainable basis in the changing climatic era.

## 1. Introduction

Plants are sessile in nature and have to face and survive in challenging abiotic (low or high temperature, excessive or inadequate light, ultraviolet radiation, drought, flood, salinity, nutrient deficiency or toxicity, and heavy metals) [1] and biotic (viruses, fungi, bacteria, nematodes, sucking and chewing pests, and herbivores) [2] stress environments with their defensive mechanisms by sensing the stress motivators and adopting some changes in their molecular system [3]. These changes are governed by a complex regulatory system through epigenetic mechanisms, which are responsible for appropriate plant reactions to drastic environmental conditions [4]. Plants often remember a specific environmental stress that they experienced during their life [5]. This ability of plants is called plant memory or epigenetic memory, which provides a smart basis for a strong and quick response to such future challenges [6].

**Figure 1:**
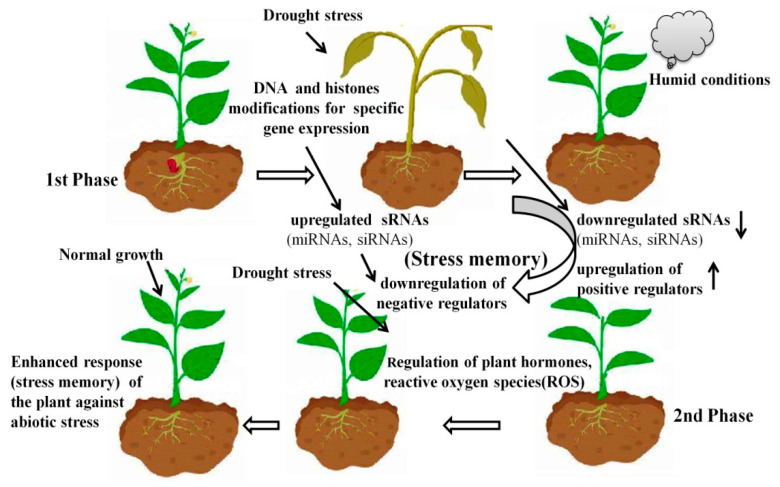
A plant exposed to abiotic stress memorizes the event and, in the 2nd phase, can resist the stress through stress memory which enhances the stress resistance of plants through DNA and histone modifications to up-regulate small RNAs (micro-RNAs (miRNAs)) and short interfering RNAs (siRNAs) and to downregulate the negative regulators (specific protein and DNA (repressor) inhibiting transcription), and through downregulation of sRNAs for the up-regulation of positive regulators (specific protein (activator) required for transcription and DNA-bound activators for transcription regulation) and regulation of hormones and reactive oxygen species (ROS).

Plant memory, specifically, “stress memory”, is an important trait that involves some specific mechanisms such as stable DNA methylation, which leads to an improved stress tolerance response in plants that have previously undergone acclimation and hardiness to the same stress driver [7]. These memories are due to epigenetic changes and play an important role by enabling plants to perform better against similar environmental hazards in the future [8]. It has been found that plants not only adopt changes for the prevailing stress scenario but also remember this information for the next generations to efficiently cope with such environmental conditions [6,9]. Epigenetic modifications provide heritable changes in the activities of genes and play an important role in gene expression to cope with environmental stresses through small RNA, expression of miRNA or siRNA for post-transcriptional gene silencing, DNA methylation, and histone changes [10,11,12,13,14,15]. The enzymes responsible for epigenetic changes are not involved directly; however, proteins as transcriptional factors and others play a mediatory role in the function of the enzymes in gene regulation to achieve the target [16]. Sometimes, the temporary arrangements to cope with the stressed environment are not stable, and as soon as the stress is over, plants behave like normal, while many research findings have shown that some modifications due to environmental stress are stable, remembered by plants, and carried over to the next generations as a stress memory [10,15,17,18]. As shown in Figure 1, a plant under drought stress during its first exposure remembers it as a stress memory and behaves normally or more efficiently at a later growth stage when facing a similar stress [15,19,20,21].

Stress memory can also be described as a mechanism to enhance the resilience of crop plants [22], and the accumulation and changes in proteins (structural and regulatory) as a transcription, translation, and transduction, which play an important role in the growth, development, and memory mechanisms of plants for stress resistance [17,23,24,25]. As the epigenetic modifications are environmentally accelerated, the phenotypic changes are mostly a reflection of a specific environmental interaction, and the changes adopted by the plant for a specific period may become permanent and heritable for future generations [19,26].

## 2. Stress Memory Enhances Abiotic Stress Resistance of Plants and Their Offspring

The stress memory induced in an early stage of plant growth can be classified as short-term stress memory or long-term stress memory. Short-term stress memory allows plants to remain resistant to a certain stress for up to about 10 days. This effect is mainly due to temporary changes in morphology and biochemical metabolites, and when the stress is over, the plants return to their previous growth status by forgetting the stress event [7]. However, long-term stress memory, which is regulated by epigenetics, could potentially last for the whole life of the plant suffering the stress and may be transferred to offspring [4,27,28]. This long-term stress memory can play an important role in the adaptation and evolution of plants to the environment through epigenetic modifications in the successor. For example, long-term stress memory enhances drought resistance in the eucalyptus (*Catalpa bungei*) [29], high-temperature resistance in the tall fescue (*Festuca arundinacea Schreb*) [30], and salt resistance in the tomato (*Solanum lycopersicum*) and ryegrass (*Lolium perenne*) [31,32]. Heat stress during the jointing stage of winter wheat can significantly increase antioxidant synthesis and photosynthetic efficiency during the grain-filling stage, which in turn enhances heat resistance and reduces the yield loss of the plants that grow out of those seeds (Table 1) [33]. Drought treatment of potatoes, at the seedling stage or tuber enlargement stage, can enhance drought resistance during the late growth stages [34,35] and it can also increase the yield under favorable growth conditions [36].

Abiotic stress in plants enhances stress resistance through stress memory in plants by up- and downregulated sRNAs (miRNAs, siRNAS) to downregulate negative regulators, to up-regulate the positive regulators, and for regulation of plant hormones, reactive oxygen species (ROS), and transcriptional factors (Figure 1) [89,90]. Abiotic stress can induce the transgenerational stress memory, and it also enhances the stress resistance of the offspring that even did not suffer the stress treatment (Table 2) [27,91,92,93,94]. Semi-lethal high-temperature treatment on wheat at the flowering stage can significantly increase the heat resistance of its subsequent generations [33]. In another study on wheat, drought priming in the first, second, and third generations resulted in a higher grain yield, leaf photosynthetic rate, and antioxidant capacity, as well as a lower O_2_ release rate and contents of H_2_O_2_ and MDA during grain filling in the fourth generation, compared to non-primed plants, and there was also a higher leaf water potential and content of proline, glycine betaine, pyrroline-5-carboxylate synthetase, and betaine aldehyde dehydrogenase in all subsequent generations of drought-primed plants [93]. A heat tolerance study of the F3 generation of *Arabidopsis* showed heat resistance in the next-generation plants, even when the heat stress was only applied to the parent and the F1 generation [95]. Similarly, in a study on potato stress resistance, it was evaluated that growing potatoes under long-term stress conditions not only contributes towards a better water-holding capacity through stress memory but also enhances the tuber yield under favorable growing conditions, irrespective of the genotype [22,36,75,76]. Previous studies on stress priming on crops including chickpea (*Cicer arietinum*), canola (*Brassica napus*), okra (*Abelmoschus esculentus*), sugarcane (*Saccharum officinarum*), maize (*Zea Mays*), and potato (*Solanum tuberosum*) provide a potential approach to improve the stress tolerance of crop plants by priming parental lines (Table 1).

## 3. Memory Induced by One Type of Stress Can Increase Cross-Resistance and Transgenerational Memory

Plants exposed to one type of stress may show a prompt response for cross-resistance against multiple and varied stresses at the intergenerational and transgenerational levels [110,111]. In both types of stress (biotic and abiotic), plants use common types of biological pathways and signals that lead to complex defensive mechanism activation at different structural levels [112]. Plants have evolved to not only survive and resist a single type of stress but to also have the ability to counter different types of environmental stresses simultaneously. Preliminary stress exposure provides plants with a better response in the future, repeated exposure confers stress priming, and epigenetic traits are mostly dominant and transmissible to next generations [9,113]. For example, drought treatment of winter wheat plants can not only increase the drought stress resistance after flowering but it can also increase crop resistance against heat; in addition, the yield and photosynthetic and antioxidant capacity were also improved in the next progeny after the parental line was exposed to heat stress [99,114].

In some cases, while encountering the stress, there may be a loss of plant vigor and yield, and it may be variable in the second stress; however, this depends upon the stability of the chromatin-modified memory genes and environmental conditions [111]. High salt-treated tomato seeds or seedlings can significantly improve the resistance of the tomato to both salt and drought stresses [31]. Drought-stressed wheat during the late growth stage can also increase plant resistance to low temperature through adjustments in photosynthesis and antioxidants [93,115,116], drought, and heat [117]. Drought treatment can also induce frost resistance in *Arabidopsis*, wheat, oats, Norway spruce, ryegrass, and strawberries [97,98,117,118,119,120]. In addition, other cross-resistance was also reported in rice, canola, *Arabidopsis*, tobacco, radish, alfalfa, potato, etc. [5,17,36,41,57,98,99,102,113,121,122,123] (Table 2). The understanding and systematic research application of the stress memory and its regulatory mechanism may provide a practical basis for the genetic improvement of plants for stress tolerance at the intergenerational and transgenerational levels. Different epigenetic modifications in the genome of chromatin states, lipids, proteins, mRNA, and hormones facilitate transgenerational memory [124].

## 4. Stress Memory Is Mainly Regulated by Epigenetic Pathways

Plant stress memory is accomplished by a cooperative regulation of physiological, translational, transcriptional, and epigenetic activities upon stress conditions [15,32]. These regulatory processes can occur at any stage of plant growth and are mainly controlled under epigenetic modifications to phenotypically reshape for environmental stress [51,125]. Due to intensive breeding, genetic diversity has become limited, and now diversity through epigenetic variations has emerged as an alternative and good source for genetic improvement of crops [125]. There have been many developments for the quantification of epigenetic variations and their impact on the growth and development of plants, leading to improved yield and quality, and, ultimately, and this has opened another avenue for breeders to breed desirable agronomic characters successfully [126]. In epigenetic modifications, DNA methylation plays an important role in gene regulation, expression, and stabilization [127]. Various enzymes (DNA methyltransferase), targeted under different plant regulatory pathway systems, take part in the process to catalyze DNA methylation for a better and quicker response against biotic and abiotic stresses [128]. Epigenetic modification has the ability to memorize the event over a long time as a plant molecular memory and the ability to respond rapidly with heritable phenotypic characteristics as an inheritance system against environmental fluxes. Some extreme abiotic stress treatments can lead to plant genome reorganization [9,129], but there are few reports indicating that short-term stress causes a large number of genomic mutations [111,130]. More evidence supports the speculation that plant stress memory is mainly regulated by epigenetic pathways [131,132], which means changing the expression pattern of the entire genome to form a rebalanced genome expression system, without changing the genome sequence [133,134].

Epigenetic regulation usually includes multiple changes to regulate heritable gene expression through post-translational and post-transcriptional changes such as phosphorylation, ubiquitination, sumolyation, DNA methylation, RNA interference, histone post-translational modifications, functional proteins, and chromatin modifications [16,135]. However, the evidence that histone modification plays a key role in the phenotypic cross-generational inheritance is sparse, and most studies have focused on the genetic mechanism of genomic DNA methylation and its correlation with phenotype [136]. DNA methylation, as an epigenetic phenomenon, means that under the action of methylase, the DNA sequence of genes is not changed, but the function of genes is changed in response to external environmental stimuli. This change is usually inherited by future generations to form epigenetic memory, which provides the possibility of breeding new stress-resistant varieties. Plant DNA methylation usually occurs at CG, CNG (N stands for any base), and CHH (H stands for A, C, T) [137]. The level of DNA methylation and its occurrence are mainly affected by the combination of various DNA methyltransferases. There are four types of methyltransferase widely found in plants. The first is maintenance methyltranferase (MET1), whose main role is to maintain the methylation of CG sites [138]. The second is domains rearranged methyltransferase (DRM), which can de novo methylate all cytosine-containing sequences and is responsible for maintaining the asymmetric CHH site [139]. The third type is chromomethylase (CMT), which is unique to plants, participates in DNA modification of heterochromatin, and can maintain the methylation of CNG sites [140,141]. The fourth type may be a homologue of the DNMT2 (DNA methyltransferase 2) family, which is conserved in many species, but its function is currently unclear [142]. Presently, many studies have reported the effects of abiotic stress on plant DNA methylation, mainly including drought, low temperature, salt, and heavy metal stress. Under stress conditions, DNA methylation can regulate the expression of stress-responsive genes [19]. In rapeseed (*Brassica napus varoleifera*) and cotton (*Gossypium hirsutum*), the number of demethylated sites in salt-tolerant lines is greater than that in sensitive lines, while the number of methylated sites is less than that in sensitive lines [143,144]. This indicates that demethylation can promote the expression of stress-related genes and is one of the important reasons for the salt resistance of rapeseed and cotton. After maize seedlings were treated with a low temperature (4 °C), the root tissue genome and cold stress-inducible gene *ZmM1* were demethylated, and the hypomethylation status caused by cold stress still did not return to normal levels after reheating at 23 °C for 7 days [145]. The hypomethylation may be related to resistance to cold stress. Similarly, low-temperature treatment also leads to a decrease in the methylation level of strawberries (*Fragaria ananassa*) and snapdragon (*Antirrhinum majus*) [146,147,148].

**Figure 2:**
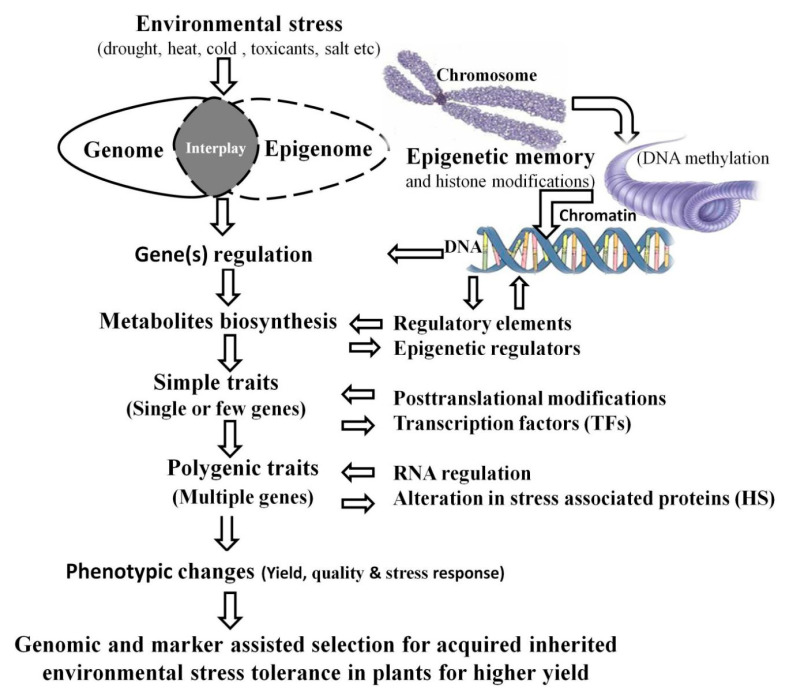
Epigenetic stress memory and selection/breeding of desired phenotypic traits of crops for stress resistance at different physiological stages from simple to complex polygenic traits through marker-assisted and genomic selection. Different regulatory factors such as plant growth hormones or epigenetic regulators, transcriptional factors, RNA regulation, small open reading frames (ORF), and histone proteins affect the gene expression and epidemically modified phenotypes of plants.

Methylation changes induced by environmental stimuli can be inherited across generations. In rice, DNA methylation variation caused by nitrogen deficiency and a decrease in the methylation level caused by 5 azacytidine treatment can be inherited for at least three generations [149,150]. DNA methylation changes caused by low-dose laser irradiation in sorghum (*Sorghum bicolor*) can also be passed onto future generations [151]. There is evidence that salt stress treatment of induced stress memory in *Arabidopsis* is achieved by regulating the transcription level of light-induced proline synthetase (P5CS1) [5]. Salt stress significantly altered the genome methylation level and gene expression patterns in *Arabidopsis*, and most of the changes were transferred stably to the next generation [152]. Some other studies on *Arabidopsis*, orchids, and rice have revealed that long-term stresses such as salt, high temperature, nitrogen fertilizer, and drought-induced changes in DNA methylation can be transferred together with stress memory (improvement in stress tolerance) to multiple generations of offspring [4,153,154,155]. Compared to the model plant, there are only some preliminary studies on stress memory in potatoes. In the study of short-term stress memory [35] or long-term stress memory [36] of potatoes, the variation in metabolism signals such as ABA, anthocyanins, antioxidants, and heat shock proteins might be affected by the change in the chromosome structure and stress-induced gene expression variation. However, it is unknown whether potato stress memory is just a short-term physiological stress response [35,156], or a long-term memory that can be stably inherited and regulated by the epigenetic pathway [36]. A significant number of studies have attempted to explain the mechanism of crop stress memory formation, from the physiological and biochemical perspectives, in order to improve stress resistance in crops, to epigenome modification. In contrast, studies at the molecular level of the epigenetic effect are scarce [157]. Therefore, if we plan to use stress memory in modern crop breeding for desired phenotypic characteristics selection, especially in multi-ploidy crops such as the potato, the molecular mechanism of stress memory should be considered for selection in the breeding process (Figure 2) [158,159]. Application of modern epigenetic tools may also be fascinating for creating new epialleic variants through editing of DNA methylation and chromatin for crop improvement by the epigenetic engineering of plants [136].

## 5. Application of Novel Plant Breeding Tools to Promote Abiotic Stress Resistance in Crops

The molecular understanding of plant responses to multiple abiotic stresses has been a hotspot among researchers for decades. The number of genes/regulatory networks, epigenetic modifications, and pathways has been studied through employing various classical, traditional biotechnological, and modern genome editing approaches. Developing abiotic stress-resistant plants in model and non-model plant species requires novel plant breeding techniques (NPBTs) for sustainable crop production [160]. The availability of genome sequencing data and multi-OMICs, along with genome editing techniques, can work in an integrative manner to open new avenues of research in breeding for abiotic stress tolerance. Plant stress memory can be further exploited through genome editing technologies to understand the underlying regulatory mechanisms. Abiotic stress in quantitative traits is controlled by multiple genes. The significant interaction between various molecular mechanisms and signaling, regulatory, and metabolic pathways for abiotic stress response/adaptation ultimately allows NPBTs to develop plants with improved traits. The presence of natural genetic variability among the same species of crop plants is integral; however, the lack in availability of diverse germplasms, screening of large numbers of mutants, cost-effectiveness and non-targeted mutation allow NPBT application in both basic and applied research [161]. NPBTs, especially genome editing technologies, have demonstrated robustness and versatility in different biological contexts [162]. The developments in genome editing technologies, along with different versions, i.e., base and prime editing, are further expanding the toolkit of genome engineering.

The application of catalytically inactive Cas9 (dCas9) has been successfully utilized in the disruption of gene function via clustered regularly interspaced short palindromic repeats interference (CRISPRi) [163]. The fusion of some effector domains, i.e., KRAB/SID, with dCas9 helped to significantly improve transcriptional repression [164]. Moreover, the paired Cas9 fusion of a transcriptional active domain, i.e., VP16/VP64, with dCas9 can activate the expression of a gene/genes of interest that allows the screening of genotypes for abiotic stress tolerance. Similarly, the synthetic transcriptional modulation of activators or repressors through CRISPR has been successfully undertaken in plants [165]. The binding of dCas9 with epigenetic modifiers assists in determining the potential role of methylation for several abiotic stress adaptations/responses in plants. The utilization of the CRISPR/Cas9 system has been employed to enable for spatial and precise modification to avoid unenviable pleiotropic effects. The promoter of the *OsRAV2* gene, a transcription factor involved in the response to saline stress, was modified with the CRISPR/Cas system to unearth the function of a specific region of *OsRAV2* promoter *GT-1*. The mutant lines showed retarded growth under salinity stress and confirmed the importance of *GT-1* for the normal functioning *OsRAV2* [166]. To unearth the potential role of osmotic stress/ABA-activated protein kinase 2 (*OsSAPK2*) for drought stress, *sapk2* mutants were generated through CRISPR technology, resulting in drought mutants in rice [167]. Moreover, the advanced toolkit of CRISPR i.e., base editing, prime editing, availability of various types of vectors, developments in gene transformation techniques, genomic sequence availability of various crop species, and free transgene editing, has made genome engineering the first and most reliable choice among researchers. Based on proof of concept in model and non-model species, it can be assumed NPBTs are the future of crop breeding [168].

## 6. Future Perspective for the Potential Application of Stress Memory for Genetic Improvement of Crops

Crop breeding for improvement in agronomic characteristics has always been linked with germplasm diversity. The variable desired traits are harbored through artificial means through crossing and selection for genetic improvement; however, the declining diversity has made it necessary to find new ways to ensure food security on a sustainable basis [13]. Breeding, marker-assisted selection, and development of genetically modified organisms/plants through biotechnology are the main ongoing research focus for crop improvement for multiple traits. Presently, epigenetics is arising as an alternative potential research approach because variants produced through epigenetic changes may be a good source for breeding and selection of crops and especially for clonal propagules [13,14]. Based on traditional breeding principles, selecting epigenetic phenotypes can be useful for breeding towards stress-tolerant plants at different levels (metabolites, simple traits, and polygenic). In this selection process, regulatory and epigenetic factors play an important role in gene expression and multi-trait development for the yield and quality of the genotypes [40,159] (Figure 2). In many recent studies, it has been found that plants have the ability to memorize past events, which can help scientists breed crops more precisely to face environmental stresses more successfully [169].

Stress priming helps plants to gain stress resistance. Verkest et al. [40] found that continuous screening under drought conditions of three generations of *Brassica napus* allowed the selected plants to gain strong drought resistance, as well as a high nitrogen utilization efficiency. Moreover, developing recombined inbred lines by mating parental lines with large epigenetic differences can be used in breeding for disease resistance or high yield [170,171]. However, compared with the model plant *Arabidopsis*, this method has not been developed for other crops [30,62,172]. Epigenetic variants can be produced through chemical treatment (5-azacytidine), epigenome editing (TALENs), zinc finger nucleases (ZFNs), and the CRISPR/Cas system to counter biotic and abiotic stresses. There is an immense amount of care needed because targeted genes may be involved in complex and multiple pathways, which may cause complex and unexpected pleiotropic effects [59,173]. All of these methods have tremendous scope in the use of epibreeding techniques for the creation of epigenetic variants for traits of interest [14]. For successful breeding through epigenetic memory, it is necessary that variations should be inherited. DNA methylation changes and histone modifications are often reset during meiosis, meaning stable inheritance of the epigenetic mark is a problem in successful breeding goals achievement [174]. In the case of clonally propagated crops such as the potato, there is an added advantage that plants do not undergo meiosis and gametogenesis, meaning the transfer of epigenetic marks through mitosis is stable. There is evidence of stable transmission of global demethylation associated epigenetic changes in up to five generations of clonally propagated *Trifolium repens* and *Fragaria vesca* for drought, soil contamination, shading, and early flowering [175,176]. Selection of epigenetic phenotypes based on the principles of traditional breeding can be potentially applied for stress tolerance development at different levels (metabolites, simple traits, and polygenic). In this selection process, regulatory and epigenetic factors play an important role in gene expression and multi-trait development of yield and quality improvements in crops [40,159] (Figure 2). Plants are able to memorize past events, which will help scientists to breed crops more precisely to face environmental stresses more efficiently [169].

Vegetatively propagated crops are efficient in the production of genotypes with added advantage for stable combinations of genes that might be lost during the sexual reproduction process; thus, clonal propagation of epigenetic variants can assure stable epigenomes with traits of interest for future generations [136]. Characterization of epigenetic variants for a large scale of the population is necessary for epibreeding [177]. Selection of epigenomes may be assisted through molecular epigenetic markers associated with phenotypic traits for precision, and the implication of these new breeding tools may broaden the scope for the development of stress-tolerant tetraploid and other crops in the shortest period of time with precision and multiplex novel traits.

## 7. Conclusions

A decline in crop yields due to biotic and abiotic stresses has been a major challenge for staple food crops, and it is estimated that the global food demand will outpace the genetic gain in the near future. Environmental changes pose significant risks to food security. Therefore, integrated solutions are required to address these challenges and increase crops yield to ensure food security. Application of epigenetic tools holds tremendous potential for improving crop varieties in terms of rapid plant adaptation, yield, and other agronomic factors to meet progressive living standards and food demands in today’s world through the production of new epialleles, including DNA methylation, histone modifications, regulation of transgenic expression, and RNA interference (RNAi). Epigenetic modifications can lead to altered gene transcription and are an important mechanism for controlling gene expression during development in response to stimulation of the environment. This epigenetic information reflects the transcriptional memory associated with cell fate decisions, developmental changes, or stress responses: memory that is often required during reproduction to be deleted and reset. In order to survive in stressful environmental conditions, plants undergo epigenetics. Such changes in plants are due to “plant stress memory”, which helps plants to react to stresses in a challenged environment and future generations to deal with stresses. Research on the relationship between epigenetics and abiotic interactions has great potential to provide answers to pressing questions about phenotypic plasticity and crop improvement. New methods of the kind discussed here and the development of new technologies will definitely increase our understanding of currently established epigenetic factors and chromatin modifications and will promote the understanding of their functions in interactions, host stresses, and crop productivity. Reprogramming by epigenetic modification caused by various environmental challenges can lead to phenotypic diversity of crops and to protection against these challenges. Development of stress-resistant crops with a high yield capacity has been the key concern of breeders in crop enhancement programs. Basic mechanistic studies of these modifications will provide proper insight into the different genes and the particular regions within them that are responsible for adapting to different abiotic stresses, leading to a better understanding of the pathway to be targeted for crop improvement.

## Figures and Tables

**Table 1:** Intergenerational stress memory resistance development in crop plants through epigenetic modifications.

Crop Species	Stress Resistance	Treatment/Pathway	References
Chickpea (*Cicer arietinum*)	Drought	Water and osmotic stress	[37,38]
Canola (*Brassica napus*)	Salt/drought	NaCl priming of seeds, halo-tolerant plant growth-promoting rhizobacteria (PGPR), increased energy use efficiency	[39,40,41]
Okra (*Abelmoschus esculentus*)	Low temperature	Solid matrix priming (SMP)	[42]
Sugarcane (*Saccharum officinarum*)	Drought/salinity	Seed (plants) priming with NaCl and PEG, drought stress memory	[21,25,43,44,45]
Mung bean (*Vigna radiate*)	Drought/salinity	Halopriming of seeds with NaCl and PEG	[46]
Maize (*Zea Mays*)	Low temperature/salt	Water, cold, NaCl, osmotic and hormonal stress	[47,48,49,50,51]
Sorghum (*Sorghum bicolar*)	Salinity	Salt priming of seedlings	[52]
Spinach (*Spinacia oleracea*)	Low temperature	Osmopriming	[49]
Rice (*Oryza japonica/indica*)	Low/high temperature/drought/salt	ABA and H_2_O_2_, salt, hydro/dehydro, osmotic, spermidine treatment of seedlings, DNA methylation, gene expression and smRNA and multi-generation drought imposition for abiotic stress tolerance	[53,54,55,56,57,58,59]
Alfalfa (*Medicago sativa*)	Drought	Seed osmotic treatment with PEG	[60]
Wheat (*Triticum aestivum*)	Drought/thermo	Water and osmotic seed priming, pre-drought/heat stress, exogenous ABA application	[33,61,62,63,64]
Cotton (*Gossypium hirsutum*)	Drought/salt/cold	NaCl and PEG treatment/miRNAs/lncRNAs expression, cold stress, DNA methylation	[65,66,67,68,69,70]
Tea plant (*Camellia sinensis*)	Drought	PEG and exogenous ABA-treated	[71]
Cowpea (*Vigna unguiculata*)	Drought	Water, osmotic, and hormonal seed stress	[72,73]
Potato (*Solanum tuberosum*)	Drought/low temperature	Long-term water stress memory, drought, and low temperature	[36,74,75,76]
Coffee (*Coffea canephora*)	Drought	Transcriptional memory	[77]
*Arabidopsis* (*Arabidopsis thaliana*)	Drought/salinity/biotic stress	β-amino-butyric acid, hyperosmotic priming of seedlings	[78,79]
Tomato (*Lycopersicon esculentum*)	Salinity/abiotic stresses	Priming of seeds with NaCl, ABA, and mannitol induced nitric oxide stresses	[80,81]
Soybean (*Glycine max*)	Drought/salt	Indole acetic acid, NaCl stress on seedlings induced long non-coding RNAs and DNA methylation	[82,83,84]
Mung bean (*Vigna radiate*)	Drought/heavy metals	Indole-3-butyric acid	[85]
Strawberry (*Fragaria ananassa*)	Salinity and non-ionic osmotic/thermotolerance	Hydrogen sulfide/sodium hydrosulfide/hydrogen peroxide and sodium nitroprusside priming of roots	[86,87,88]

**Table 2:** Transgenerational/cross-stress resistance development in crop plants through stress memory/epigenetic modifications.

Crop Species	Stress Resistance	Primary Exposure/Treatment	Transgenerational Physical Response	References
*Arabidopsis* (*Arabidopsis thaliana*)	Drought, extreme light adoption	β–aminobutyric acid (BABA), dehydration stress, salt and heat stress, short wavelength radiations	Descendants exhibit biotic and abiotic stress resistance, phenotypic changes for increased flexibility	[5,9,78,96,97,98]
Wheat (*Triticum aestivum*)	Drought/salt/heat	Terminal drought/water, osmotic and heat priming of first-generation plants	Drought memory improved resistance against salt stress and drought, and thermotolerance	[67,99,100]
Canola (*Brassica napus*)	Cold/heat/drought	Cold acclimation	Heat/drought resistance, increased growth and yield	[101,102]
Rice (*Oryza sativa*)	Abiotic	Heavy metals, sublethal heat exposure, drought	Enhanced tolerance through heritable changes in gene expression and DNA methylation	[4,92,103]
Maize (*Zea mays*)	Drought/salt	Osmotic stress	Through epigenetic mechanisms, better response to abiotic stresses	[104]
Tomato (*Lycopersicon sculentum*)	Cold	Hydrogen peroxide pretreatment of roots, arginase induction by heat treatment of fruit	Enhanced oxidative stress response, amelioration of chilling injury, and activation of antioxidant enzymes	[105,106]
Turnip/Field mustard (*Brassica rapa*/*campestris*)	Heat/cold shock/biotic	Heat/salinity/drought/biotic	Stress-induced transgenerational inheritance and cross-protection	[107,108]
Pea (*Pisum sativum*)	Heavy metals	Acclimated to low temperature	Cold-induced photo-inhibition	[109]
Alfalfa (*Medicago sativa*)	Drought	Drought stress/osmopriming	Enhanced growth under drought	[60]

## References

[1] Pereira A. (2016). Plant Abiotic Stress Challenges from the Changing Environment. Front. Plant Sci..

[2] Porter S.S., Bantay R., Friel C.A., Garoutte A., Gdanetz K., Ibarreta K., Moore B.M., Shetty P., Siler E., Friesen M.L. (2020). Beneficial microbes ameliorate abiotic and biotic sources of stress on plants. Funct. Ecol..

[3] Zhang Y., Lv Y., Jahan N., Chen G., Ren D., Guo L. (2018). Sensing of Abiotic Stress and Ionic Stress Responses in Plants. Int. J. Mol. Sci..

[4] Zheng X., Chen L., Xia H., Wei H., Lou Q., Li M., Li T., Luo L. (2017). Transgenerational epimutations induced by multi-generation drought imposition mediate rice plant’s adaptation to drought condition. Sci. Rep..

[5] Feng X.J., Li J.R., Qi S.L., Lin Q.F., Jin J.B., Hua X.J. (2016). Light affects salt stress-induced transcriptional memory of P5CS1 in Arabidopsis. Proc. Natl. Acad. Sci. USA.

[6] Latzel V., Rendina González A.P., Rosenthal J. (2016). Epigenetic Memory as a Basis for Intelligent Behavior in Clonal Plants. Front. Plant Sci..

[7] Crisp P.A., Ganguly D., Eichten S.R., Borevitz J.O., Pogson B.J. (2016). Reconsidering plant memory: Intersections between stress recovery, RNA turnover, and epigenetics. Sci. Adv..

[8] Ding Y., Fromm M., Avramova Z. (2012). Multiple exposures to drought ’train’ transcriptional responses in Arabidopsis. Nat. Commun..

[9] Molinier J., Ries G., Zipfel C., Hohn B. (2006). Transgeneration memory of stress in plants. Nature.

[10] Thiebaut F., Hemerly A.S., Ferreira P.C.G. (2019). A Role for Epigenetic Regulation in the Adaptation and Stress Responses of Non-model Plants. Front. Plant Sci..

[11] Wojtyla Ł., Paluch-Lubawa E., Sobieszczuk-Nowicka E., Garnczarska M. (2020). Drought Stress Memory and Subsequent Drought Stress Tolerance in Plants. Priming-Mediated Stress and Cross-Stress Tolerance in Crop Plants.

[12] Liew Y.J., Howells E.J., Wang X., Michell C.T., Burt J.A., Idaghdour Y., Aranda M. (2020). Intergenerational epigenetic inheritance in reef-building corals. Nat. Clim. Chang..

[13] Latutrie M., Gourcilleau D., Pujol B. (2019). Epigenetic variation for agronomic improvement: An opportunity for vegetatively propagated crops. Am. J. Bot..

[14] Kapazoglou A., Ganopoulos I., Tani E., Tsaftaris A., Kuntz M. (2018). Chapter Nine—Epigenetics, Epigenomics and Crop Improvement. Advances in Botanical Research.

[15] Kinoshita T., Seki M. (2014). Epigenetic memory for stress response and adaptation in plants. Plant Cell Physiol..

[16] Santos A.P., Serra T., Figueiredo D.D., Barros P., Lourenço T., Chander S., Oliveira M.M., Saibo N.J.M. (2011). Transcription Regulation of Abiotic Stress Responses in Rice: A Combined Action of Transcription Factors and Epigenetic Mechanisms. OMICS J. Integr. Biol..

[17] Bruce T.J.A., Matthes M.C., Napier J.A., Pickett J.A. (2007). Stressful “memories” of plants: Evidence and possible mechanisms. Plant Sci..

[18] Munne-Bosch S., Alegre L. (2013). Cross-stress tolerance and stress “memory” in plants. Environ. Exp. Bot..

[19] Chinnusamy V., Zhu J.-K. (2009). Epigenetic regulation of stress responses in plants. Curr. Opin. Plant Biol..

[20] Tombesi S., Frioni T., Poni S., Palliotti A. (2018). Effect of water stress “memory” on plant behavior during subsequent drought stress. Environ. Exp. Bot..

[21] Marcos F.C.C., Silveira N.M., Marchiori P.E.R., Machado E.C., Souza G.M., Landell M.G.A., Ribeiro R.V. (2018). Drought tolerance of sugarcane propagules is improved when origin material faces water deficit. PLoS ONE.

[22] Walter J., Nagy L., Hein R., Rascher U., Beierkuhnlein C., Willner E., Jentsch A. (2011). Do plants remember drought? Hints towards a drought-memory in grasses. Environ. Exp. Bot..

[23] Janmohammadi M., Zolla L., Rinalducci S. (2015). Low temperature tolerance in plants: Changes at the protein level. Phytochemistry.

[24] Ali A., Khan M., Sharif R., Mujtaba M., Gao S.-J. (2019). Sugarcane Omics: An Update on the Current Status of Research and Crop Improvement. Plants.

[25] Marcos F.C.C., Silveira N.M., Mokochinski J.B., Sawaya A.C.H.F., Marchiori P.E.R., Machado E.C., Souza G.M., Landell M.G.A., Ribeiro R.V. (2018). Drought tolerance of sugarcane is improved by previous exposure to water deficit. J. Plant Physiol..

[26] Verhoeven K.J.F., Jansen J.J., van Dijk P.J., Biere A. (2010). Stress-induced DNA methylation changes and their heritability in asexual dandelions. New Phytol..

[27] Bilichak A., Kovalchuk I. (2016). Transgenerational response to stress in plants and its application for breeding. J. Exp. Bot..

[28] Vialou V., Feng J., Robison A.J., Nestler E.J. (2013). Epigenetic mechanisms of depression and antidepressant action. Annu. Rev. Pharm. Toxicol..

[29] Zheng H., Zhang X., Ma W., Song J., Rahman S.U., Wang J., Zhang Y. (2017). Morphological and physiological responses to cyclic drought in two contrasting genotypes of Catalpa bungei. Environ. Exp. Bot..

[30] Hu T., Liu S.Q., Amombo E., Fu J.M. (2015). Stress memory induced rearrangements of HSP transcription, photosystem II photochemistry and metabolism of tall fescue (Festuca arundinacea Schreb.) in response to high-temperature stress. Front. Plant Sci..

[31] Cuartero J., Bolarin M.C., Asins M.J., Moreno V. (2006). Increasing salt tolerance in the tomato. J. Exp. Bot..

[32] Hu T., Jin Y., Li H., Amombo E., Fu J. (2016). Stress memory induced transcriptional and metabolic changes of perennial ryegrass (Lolium perenne) in response to salt stress. Physiol. Plant..

[33] Wang X., Cai J., Liu F., Dai T., Cao W., Wollenweber B., Jiang D. (2014). Multiple heat priming enhances thermo-tolerance to a later high temperature stress via improving subcellular antioxidant activities in wheat seedlings. Plant Physiol. Biochem..

[34] Yactayo W., Ramírez D.A., Gutiérrez R., Mares V., Posadas A., Quiroz R. (2013). Effect of partial root-zone drying irrigation timing on potato tuber yield and water use efficiency. Agric. Water Manag..

[35] Watkinson J.I., Hendricks L., Sioson A.A., Vasquez-Robinet C., Stromberg V., Heath L.S., Schuler M., Bohnert H.J., Bonierbale M., Grene R. (2006). Accessions of Solanum tuberosum ssp. andigena show differences in photosynthetic recovery after drought stress as reflected in gene expression profiles. Plant Sci..

[36] Ramírez D.A., Rolando J.L., Yactayo W., Monneveux P., Mares V., Quiroz R. (2015). Improving potato drought tolerance through the induction of long-term water stress memory. Plant Sci..

[37] Elkoca E., Haliloglu K., Esitken A., Ercisli S. (2007). Hydro- and osmopriming improve chickpea germination. Acta Agric. Scand. Sect. B Soil Plant Sci..

[38] Kaur S., Gupta A.K., Kaur N. (2002). Effect of osmo- and hydropriming of chickpea seeds on seedling growth and carbohydrate metabolism under water deficit stress. Plant Growth Regul..

[39] Farhoudi R., Sharifzadeh F., Poustini K., Makkizadeh M.T., Kochak Por M. (2007). The effects of NaCl priming on salt tolerance in canola (Brassica napus) seedlings grown under saline conditions. Seed Sci. Technol..

[40] Verkest A., Byzova M., Martens C., Willems P., Verwulgen T., Slabbinck B., Rombaut D., Van de Velde J., Vandepoele K., Standaert E. (2015). Selection for Improved Energy Use Efficiency and Drought Tolerance in Canola Results in Distinct Transcriptome and Epigenome Changes. Plant Physiol..

[41] Liu Y., Han J., Chen Z., Wu H., Dong H., Nie G. (2017). Engineering cell signaling using tunable CRISPR–Cpf1-based transcription factors. Nat. Commun..

[42] Pandita V.K., Anand A., Nagarajan S., Seth R., Sinha S.N. (2010). Solid matrix priming improves seed emergence and crop performance in okra. Seed Sci. Technol..

[43] Patade V.Y., Bhargava S., Suprasanna P. (2012). Halopriming mediated salt and iso-osmotic PEG stress tolerance and, gene expression profiling in sugarcane (*Saccharum officinarum* L.). Mol. Biol. Rep..

[44] Fleta-Soriano E., Munné-Bosch S. (2016). Stress Memory and the Inevitable Effects of Drought: A Physiological Perspective. Front. Plant Sci..

[45] Khalil F., Naiyan X., Tayyab M., Pinghua C. (2018). Screening of EMS-Induced Drought-Tolerant Sugarcane Mutants Employing Physiological, Molecular and Enzymatic Approaches. Agronomy.

[46] Jisha K.C., Puthur J.T. (2014). Halopriming of seeds imparts tolerance to NaCl and PEG induced stress in *Vigna radiata* (L.) Wilczek varieties. Physiol. Mol. Biol. Plants.

[47] Tajdoost S., Farboodnia T., Heidari R. (2007). Salt pretreatment enhance salt tolerance in *Zea mays* L. seedlings. Pak. J. Biol. Sci..

[48] Tan M.-P. (2010). Analysis of DNA methylation of maize in response to osmotic and salt stress based on methylation-sensitive amplified polymorphism. Plant Physiol. Biochem..

[49] Chen K., Fessehaie A., Arora R. (2012). Selection of Reference Genes for Normalizing Gene Expression during Seed Priming and Germination Using qPCR in Zea mays and Spinacia oleracea. Plant Mol. Biol. Rep..

[50] Shan X., Wang X., Yang G., Wu Y., Su S., Li S., Liu H., Yuan Y. (2013). Analysis of the DNA methylation of maize (*Zea mays* L.) in response to cold stress based on methylation-sensitive amplified polymorphisms. J. Plant Biol..

[51] Rehman H.u., Iqbal H., Basra S.M.A., Afzal I., Farooq M., Wakeel A., Wang N. (2015). Seed priming improves early seedling vigor, growth and productivity of spring maize. J. Integr. Agric..

[52] Yan K., Xu H., Cao W., Chen X. (2015). Salt priming improved salt tolerance in sweet sorghum by enhancing osmotic resistance and reducing root Na+ uptake. Acta Physiol. Plant..

[53] Cheng C., Yun K.-Y., Ressom H.W., Mohanty B., Bajic V.B., Jia Y., Yun S.J., de los Reyes B.G. (2007). An early response regulatory cluster induced by low temperature and hydrogen peroxide in seedlings of chilling-tolerant japonica rice. BMC Genom..

[54] Gayacharan, Joel A.J. (2013). Epigenetic responses to drought stress in rice (*Oryza sativa* L.). Physiol. Mol. Biol. Plants.

[55] Goswami A., Banerjee R., Raha S. (2013). Drought resistance in rice seedlings conferred by seed priming. Protoplasma.

[56] Mostofa M.G., Yoshida N., Fujita M. (2014). Spermidine pretreatment enhances heat tolerance in rice seedlings through modulating antioxidative and glyoxalase systems. Plant Growth Regul..

[57] Wang W., Huang F., Qin Q., Zhao X., Li Z., Fu B. (2015). Comparative analysis of DNA methylation changes in two rice genotypes under salt stress and subsequent recovery. Biochem. Biophys. Res. Commun..

[58] Zhang H., Xu F., Wu Y., Hu H.-h., Dai X.-f. (2017). Progress of potato staple food research and industry development in China. J. Integr. Agric..

[59] Garg R., Narayana Chevala V., Shankar R., Jain M. (2015). Divergent DNA methylation patterns associated with gene expression in rice cultivars with contrasting drought and salinity stress response. Sci. Rep..

[60] Mouradi M., Bouizgaren A., Farissi M., Latrach L., Qaddoury A., Ghoulam C. (2016). Seed osmopriming improves plant growth, nodulation, chlorophyll fluorescence and nutrient uptake in alfalfa (*Medicago sativa* L.)—Rhizobia symbiosis under drought stress. Sci. Hortic..

[61] Du Y.L., Wang Z.Y., Fan J.W., Turner N.C., He J., Wang T., Li F.M. (2013). Exogenous abscisic acid reduces water loss and improves antioxidant defence, desiccation tolerance and transpiration efficiency in two spring wheat cultivars subjected to a soil water deficit. Funct. Plant Biol..

[62] Li X., Cai J., Liu F., Dai T., Cao W., Jiang D. (2014). Exogenous Abscisic Acid Application during Grain Filling in Winter Wheat Improves Cold Tolerance of Offspring’s Seedlings. J. Agron. Crop Sci..

[63] Lemmens E., Deleu L.J., De Brier N., De Man W.L., De Proft M., Prinsen E., Delcour J.A. (2019). The Impact of Hydro-Priming and Osmo-Priming on Seedling Characteristics, Plant Hormone Concentrations, Activity of Selected Hydrolytic Enzymes, and Cell Wall and Phytate Hydrolysis in Sprouted Wheat (*Triticum aestivum* L.). ACS Omega.

[64] Abid M., Tian Z., Zahoor R., Ata-Ul-Karim S.T., Daryl C., Snider J.L., Dai T., Sparks D.L. (2018). Chapter Two—Pre-Drought Priming: A Key Drought Tolerance Engine in Support of Grain Development in Wheat. Advances in Agronomy.

[65] Fan H.H., Wei J., Li T.C., Li Z.P., Guo N., Cai Y.P., Lin Y. (2013). DNA methylation alterations of upland cotton (*Gossypium hirsutum*) in response to cold stress. Acta Physiol. Plant..

[66] Wang M., Wang Q., Zhang B. (2013). Response of miRNAs and their targets to salt and drought stresses in cotton (*Gossypium hirsutum* L.). Gene.

[67] Wang B., Zhang M., Fu R., Qian X., Rong P., Zhang Y., Jiang P., Wang J., Lu X., Wang D. (2016). Epigenetic mechanisms of salt tolerance and heterosis in Upland cotton (*Gossypium hirsutum* L.) revealed by methylation-sensitive amplified polymorphism analysis. Euphytica.

[68] Gao S., Yang L., Zeng H.Q., Zhou Z.S., Yang Z.M., Li H., Sun D., Xie F., Zhang B. (2016). A cotton miRNA is involved in regulation of plant response to salt stress. Sci. Rep..

[69] Lu X., Chen X., Mu M., Wang J., Wang X., Wang D., Yin Z., Fan W., Wang S., Guo L. (2016). Genome-Wide Analysis of Long Noncoding RNAs and Their Responses to Drought Stress in Cotton (*Gossypium hirsutum* L.). PLoS ONE.

[70] Lu X., Wang X., Chen X., Shu N., Wang J., Wang D., Wang S., Fan W., Guo L., Guo X. (2017). Single-base resolution methylomes of upland cotton (Gossypium hirsutum L.) reveal epigenome modifications in response to drought stress. BMC Genom..

[71] Zhou L., Xu H., Mischke S., Meinhardt L.W., Zhang D., Zhu X., Li X., Fang W. (2014). Exogenous abscisic acid significantly affects proteome in tea plant (Camellia sinensis) exposed to drought stress. Hortic. Res..

[72] Eskandari H., Kazemi K. (2011). Effect of seed priming on germination properties and seedling establishment of cowpea (*Vigna sinensis*). Not. Sci. Biol..

[73] Boucelha L., Djebbar R. (2015). Influence of different pre-germination treatments of *Vigna unguiculata* (L.) Walp. seeds on germination performance and water stress tolerance. Biotechnol. Agron. Société Environ..

[74] Xin C., Hou R., Wu F., Zhao Y., Xiao H., Si W., Ali M.E., Cai L., Guo J. (2015). Analysis of cytosine methylation status in potato by methylation-sensitive amplified polymorphisms under low-temperature stress. J. Plant Biol..

[75] Banik P., Zeng W., Tai H., Bizimungu B., Tanino K. (2016). Effects of drought acclimation on drought stress resistance in potato (Solanum tuberosum L.) genotypes. Environ. Exp. Bot..

[76] Boguszewska-Mańkowska D., Pieczyński M., Wyrzykowska A., Kalaji H.M., Sieczko L., Szweykowska-Kulińska Z., Zagdańska B. (2018). Divergent strategies displayed by potato (Solanum tuberosum L.) cultivars to cope with soil drought. J. Agron. Crop Sci..

[77] Guedes F.A.d.F., Nobres P., Rodrigues Ferreira D.C., Menezes-Silva P.E., Ribeiro-Alves M., Correa R.L., DaMatta F.M., Alves-Ferreira M. (2018). Transcriptional memory contributes to drought tolerance in coffee (Coffea canephora) plants. Environ. Exp. Bot..

[78] Slaughter A., Daniel X., Flors V., Luna E., Hohn B., Mauch-Mani B. (2012). Descendants of Primed Arabidopsis Plants Exhibit Resistance to Biotic Stress. Plant Physiol..

[79] Sani E., Herzyk P., Perrella G., Colot V., Amtmann A. (2013). Hyperosmotic priming of Arabidopsis seedlings establishes a long-term somatic memory accompanied by specific changes of the epigenome. Genome Biol..

[80] Amooaghaie R., Nikzad K. (2013). The role of nitric oxide in priming-induced low-temperature tolerance in two genotypes of tomato. Seed Sci. Res..

[81] Omidvar V., Fellner M. (2015). DNA Methylation and Transcriptomic Changes in Response to Different Lights and Stresses in 7B-1 Male-Sterile Tomato. PLoS ONE.

[82] Umezawa T., Shimizu K., Kato M., Ueda T. (2000). Enhancement of salt tolerance in soybean with NaCl pretreatment. Physilogia Plant..

[83] Lecube M.L., Noriega G.O., Santa Cruz D.M., Tomaro M.L., Batlle A., Balestrasse K.B. (2014). Indole acetic acid is responsible for protection against oxidative stress caused by drought in soybean plants: The role of heme oxygenase induction. Redox Rep..

[84] Chen R., Li M., Zhang H., Duan L., Sun X., Jiang Q., Zhang H., Hu Z. (2019). Continuous salt stress-induced long non-coding RNAs and DNA methylation patterns in soybean roots. BMC Genom..

[85] Li S.-W., Zeng X.-Y., Leng Y., Feng L., Kang X.-H. (2018). Indole-3-butyric acid mediates antioxidative defense systems to promote adventitious rooting in mung bean seedlings under cadmium and drought stresses. Ecotoxicol. Environ. Saf..

[86] Christou A., Manganaris G.A., Papadopoulos I., Fotopoulos V. (2013). Hydrogen sulfide induces systemic tolerance to salinity and non-ionic osmotic stress in strawberry plants through modification of reactive species biosynthesis and transcriptional regulation of multiple defence pathways. J. Exp. Bot..

[87] Christou A., Filippou P., Manganaris G.A., Fotopoulos V. (2014). Sodium hydrosulfide induces systemic thermotolerance to strawberry plants through transcriptional regulation of heat shock proteins and aquaporin. BMC Plant Biol..

[88] Christou A., Manganaris G.A., Fotopoulos V. (2014). Systemic mitigation of salt stress by hydrogen peroxide and sodium nitroprusside in strawberry plants via transcriptional regulation of enzymatic and non-enzymatic antioxidants. Environ. Exp. Bot..

[89] Banerjee S., Sirohi A., Ansari A.A., Gill S.S. (2017). Role of small RNAs in abiotic stress responses in plants. Plant Gene.

[90] Zhang M., An P., Li H., Wang X., Zhou J., Dong P., Zhao Y., Wang Q., Li C. (2019). The miRNA-Mediated Post-Transcriptional Regulation of Maize in Response to High Temperature. Int. J. Mol. Sci..

[91] Frost C.J., Mescher M.C., Carlson J.E., De Moraes C.M. (2008). Plant defense priming against herbivores: Getting ready for a different battle. Plant Physiol..

[92] Shi W., Lawas L., Raju B., Jagadish S. (2016). Acquired thermo-tolerance and trans-generational heat stress response at flowering in rice. J. Agron. Crop Sci..

[93] Wang X., Zhang X., Chen J., Wang X., Cai J., Zhou Q., Dai T., Cao W., Jiang D. (2018). Parental Drought-Priming Enhances Tolerance to Post-anthesis Drought in Offspring of Wheat. Front. Plant Sci..

[94] Qin F., Shinozaki K., Yamaguchi-Shinozaki K. (2011). Achievements and Challenges in Understanding Plant Abiotic Stress Responses and Tolerance. Plant Cell Physiol..

[95] Whittle C., Otto S., Johnston M.O., Krochko J. (2009). Adaptive epigenetic memory of ancestral temperature regime in Arabidopsis thaliana. Botany.

[96] Ding Y., Avramova Z., Fromm M. (2011). The Arabidopsis trithorax-like factor ATX1 functions in dehydration stress responses via ABA-dependent and ABA-independent pathways. Plant J. Cell Mol. Biol..

[97] Migicovsky Z., Yao Y., Kovalchuk I. (2014). Transgenerational phenotypic and epigenetic changes in response to heat stress in Arabidopsis thaliana. Plant Signal Behav..

[98] Ganguly D.R., Crisp P.A., Eichten S.R., Pogson B.J. (2017). The Arabidopsis DNA Methylome Is Stable under Transgenerational Drought Stress. Plant Physiol..

[99] Zhang X., Wang X., Zhong J., Zhou Q., Wang X., Cai J., Dai T., Cao W., Jiang D. (2016). Drought priming induces thermo-tolerance to post-anthesis high-temperature in offspring of winter wheat. Environ. Exp. Bot..

[100] Tabassum T., Farooq M., Ahmad R., Zohaib A., Wahid A. (2017). Seed priming and transgenerational drought memory improves tolerance against salt stress in bread wheat. Plant Physiol. Biochem..

[101] Liu T., Li Y., Duan W., Huang F., Hou X. (2017). Cold acclimation alters DNA methylation patterns and confers tolerance to heat and increases growth rate in Brassica rapa. J. Exp. Bot..

[102] Hatzig S.V., Nuppenau J.-N., Snowdon R.J., Schießl S.V. (2018). Drought stress has transgenerational effects on seeds and seedlings in winter oilseed rape (*Brassica napus* L.). BMC Plant Biol..

[103] Ou X., Zhang Y., Xu C., Lin X., Zang Q., Zhuang T., Jiang L., von Wettstein D., Liu B. (2012). Transgenerational inheritance of modified DNA methylation patterns and enhanced tolerance induced by heavy metal stress in rice (Oryza sativa L.). PLoS ONE.

[104] Forestan C., Aiese Cigliano R., Farinati S., Lunardon A., Sanseverino W., Varotto S. (2016). Stress-induced and epigenetic-mediated maize transcriptome regulation study by means of transcriptome reannotation and differential expression analysis. Sci. Rep..

[105] İşeri Ö.D., Körpe D.A., Sahin F.I., Haberal M. (2013). Hydrogen peroxide pretreatment of roots enhanced oxidative stress response of tomato under cold stress. Acta Physiol. Plant..

[106] Zhang X., Shen L., Li F., Meng D., Sheng J. (2013). Arginase induction by heat treatment contributes to amelioration of chilling injury and activation of antioxidant enzymes in tomato fruit. Postharvest Biol. Technol..

[107] Bilichak A., Ilnytskyy Y., Wóycicki R., Kepeshchuk N., Fogen D., Kovalchuk I. (2015). The elucidation of stress memory inheritance in Brassica rapa plants. Front. Plant Sci..

[108] Kellenberger R.T., Desurmont G.A., Schlüter P.M., Schiestl F.P. (2018). Trans-generational inheritance of herbivory-induced phenotypic changes in Brassica rapa. Sci. Rep..

[109] Streb P., Aubert S., Gout E., Feierabend J., Bligny R. (2008). Cross tolerance to heavy-metal and cold-induced photoinhibiton in leaves of Pisum sativum acclimated to low temperature. Physiol. Mol. Biol. Plants.

[110] Foyer C.H., Rasool B., Davey J.W., Hancock R.D. (2016). Cross-tolerance to biotic and abiotic stresses in plants: A focus on resistance to aphid infestation. J. Exp. Bot..

[111] Lämke J., Bäurle I. (2017). Epigenetic and chromatin-based mechanisms in environmental stress adaptation and stress memory in plants. Genome Biol..

[112] Atkinson N.J., Urwin P.E. (2012). The interaction of plant biotic and abiotic stresses: From genes to the field. J. Exp. Bot..

[113] Wang X., Vignjevic M., Jiang D., Jacobsen S., Wollenweber B. (2014). Improved tolerance to drought stress after anthesis due to priming before anthesis in wheat (Triticum aestivum L.) var. Vinjett. J. Exp. Bot..

[114] Wang X., Xin C., Cai J., Zhou Q., Dai T., Cao W., Jiang D. (2016). Heat Priming Induces Trans-generational Tolerance to High Temperature Stress in Wheat. Front. Plant Sci..

[115] Li X., Topbjerg H.B., Jiang D., Liu F. (2015). Drought priming at vegetative stage improves the antioxidant capacity and photosynthesis performance of wheat exposed to a short-term low temperature stress at jointing stage. Plant Soil.

[116] Li X., Liu F., Hossain M.A., Wani S.H., Bhattacharjee S., Burritt D.J., Tran L.-S.P. (2016). Drought Stress Memory and Drought Stress Tolerance in Plants: Biochemical and Molecular Basis. Drought Stress Tolerance in Plants, Vol 1: Physiology and Biochemistry.

[117] Wang X., Vignjevic M., Liu F., Jacobsen S., Jiang D., Wollenweber B. (2015). Drought priming at vegetative growth stages improves tolerance to drought and heat stresses occurring during grain filling in spring wheat. Plant Growth Regul..

[118] Rajashekar C.B., Panda M.J.S.H. (2014). Water stress is a component of cold acclimation process essential for inducing full freezing tolerance in strawberry. Sci. Hortic..

[119] Kreyling J., Wiesenberg G.L., Thiel D., Wohlfart C., Huber G., Walter J., Jentsch A., Konnert M., Beierkuhnlein C. (2012). Cold hardiness of Pinus nigra Arnold as influenced by geographic origin, warming, and extreme summer drought. Environ. Exp. Bot..

[120] Blodner C., Skroppa T., Johnsen O., Polle A. (2005). Freezing tolerance in two Norway spruce (Picea abies [L.] Karst.) progenies is physiologically correlated with drought tolerance. J. Plant Physiol..

[121] Trewavas A. (2009). What is plant behaviour?. Plant Cell Environ..

[122] Vu W.T., Chang P.L., Moriuchi K.S., Friesen M.L. (2015). Genetic variation of transgenerational plasticity of offspring germination in response to salinity stress and the seed transcriptome of Medicago truncatula. BMC Evol. Biol..

[123] Choi C.S., Sano H. (2007). Abiotic-stress induces demethylation and transcriptional activation of a gene encoding a glycerophosphodiesterase-like protein in tobacco plants. Mol. Genet. Genom..

[124] Mozgova I., Mikulski P., Pecinka A., Farrona S., Alvarez-Venegas R., De-la-Peña C., Casas-Mollano J. (2019). Epigenetic Mechanisms of Abiotic Stress Response and Memory in Plants. Epigenetics in Plants of Agronomic Importance: Fundamentals and Applications.

[125] Gallusci P., Dai Z., Génard M., Gauffretau A., Leblanc-Fournier N., Richard-Molard C., Vile D., Brunel-Muguet S. (2017). Epigenetics for Plant Improvement: Current Knowledge and Modeling Avenues. Trends Plant Sci..

[126] Cortijo S., Wardenaar R., Colomé-Tatché M., Gilly A., Etcheverry M., Labadie K., Caillieux E., Hospital F., Aury J.-M., Wincker P. (2014). Mapping the Epigenetic Basis of Complex Traits. Science.

[127] Lang Z., Wang Y., Tang K., Tang D., Datsenka T., Cheng J., Zhang Y., Handa A.K., Zhu J.-K. (2017). Critical roles of DNA demethylation in the activation of ripening-induced genes and inhibition of ripening-repressed genes in tomato fruit. Proc. Natl. Acad. Sci. USA.

[128] Zhang H., Lang Z., Zhu J.-K. (2018). Dynamics and function of DNA methylation in plants. Nat. Rev. Mol. Cell Biol..

[129] Klumpp A., Ansel W., Fomin A., Schnirring S., Pickl C. (2004). Influence of climatic conditions on the mutations in pollen mother cells of Tradescantia clone 4430 and implications for the Trad-MCN bioassay protocol. Hereditas.

[130] Cruzan M.B., Streisfeld M.A., Schwoch J.A. (2018). Phenotypic Effects of Somatic Mutations Accumulating during Vegetative Growth. bioRxiv.

[131] Alsdurf J., Anderson C., Siemens D.H. (2015). Epigenetics of drought-induced trans-generational plasticity: Consequences for range limit development. AOB Plants.

[132] Tang X.-M., Tao X., Wang Y., Ma D.-W., Li D., Yang H., Ma X.-R. (2014). Genomics. Analysis of DNA methylation of perennial ryegrass under drought using the methylation-sensitive amplification polymorphism (MSAP) technique. Mol. Genet..

[133] Madlung A., Comai L. (2004). The effect of stress on genome regulation and structure. Ann. Bot..

[134] Habu Y., Kakutani T., Paszkowski J. (2001). Epigenetic developmental mechanisms in plants: Molecules and targets of plant epigenetic regulation. Curr. Opin. Genet. Dev..

[135] Hirayama T., Shinozaki K. (2010). Research on plant abiotic stress responses in the post-genome era: Past, present and future. Plant J..

[136] Springer N.M., Schmitz R.J. (2017). Exploiting induced and natural epigenetic variation for crop improvement. Nat. Reviews. Genet..

[137] Vanyushin B.F., Ashapkin V.V. (2011). DNA methylation in higher plants: Past, present and future. Biochim. Et Biophys. Acta (Bba) Gene Regul. Mech..

[138] Finnegan E.J., Kovac K.A. (2000). Plant DNA methyltransferases. Plant Mol. Biol..

[139] Matzke M., Kanno T., Huettel B., Daxinger L., Matzke A.J.M. (2007). Targets of RNA-directed DNA methylation. Curr. Opin. Plant Biol..

[140] Tompa R., McCallum C.M., Delrow J., Henikoff J.G., van Steensel B., Henikoff S. (2002). Genome-Wide Profiling of DNA Methylation Reveals Transposon Targets of CHROMOMETHYLASE3. Curr. Biol..

[141] Lindroth A.M., Cao X., Jackson J.P., Zilberman D., McCallum C.M., Henikoff S., Jacobsen S.E. (2001). Requirement of CHROMOMETHYLASE3 for Maintenance of CpXpG Methylation. Science.

[142] Goodrich J., Tweedie S. (2002). Remembrance of things past: Chromatin remodeling in plant development. Annu. Rev. Cell Dev. Biol..

[143] Marconi G., Pace R., Traini A., Raggi L., Lutts S., Chiusano M., Guiducci M., Falcinelli M., Benincasa P., Albertini E. (2013). Use of MSAP markers to analyse the effects of salt stress on DNA methylation in rapeseed (Brassica napus var. oleifera). PLoS ONE.

[144] Zhao Y.-l., Yu S.-x., Ye W.-w., Wang H.-m., Wang J.-j., Fang B.-x. (2010). Study on DNA Cytosine Methylation of Cotton (Gossypium hirsutum L.) Genome and Its Implication for Salt Tolerance. Agric. Sci. China.

[145] Steward N., Ito M., Yamaguchi Y., Koizumi N., Sano H. (2002). Periodic DNA methylation in maize nucleosomes and demethylation by environmental stress. J. Biol. Chem..

[146] Hao Y.J., You C.X., Deng X.X. (2002). Analysis of ploidy and the patterns of amplified fragment length polymorphism and methylation sensitive amplified polymorphism in strawberry plants recovered from cryopreservation. Cryo Lett..

[147] Hashida S.-n., Kitamura K., Mikami T., Kishima Y. (2003). Temperature shift coordinately changes the activity and the methylation state of transposon Tam3 in Antirrhinum majus. Plant Physiol..

[148] Hashida S.-N., Uchiyama T., Martin C., Kishima Y., Sano Y., Mikami T. (2006). The temperature-dependent change in methylation of the Antirrhinum transposon Tam3 is controlled by the activity of its transposase. Plant Cell.

[149] Kou H.P., Li Y., Song X.X., Ou X.F., Xing S.C., Ma J., Von Wettstein D., Liu B. (2011). Heritable alteration in DNA methylation induced by nitrogen-deficiency stress accompanies enhanced tolerance by progenies to the stress in rice (Oryza sativa L.). J. Plant Physiol..

[150] Fieldes M.A., Schaeffer S.M., Krech M.J., Brown J.C. (2005). DNA hypomethylation in 5-azacytidine-induced early-flowering lines of flax. Tag. Theor. Appl. Genetics. Theor. Und Angew. Genet..

[151] Wang H., Feng Q., Zhang M., Yang C., Sha W., Liu B. (2010). Alteration of DNA methylation level and pattern in sorghum (Sorghum bicolor L.) pure-lines and inter-line F1 hybrids following low-dose laser irradiation. J. Photochem. Photobiol. B Biol..

[152] Jiang C., Mithani A., Belfield E.J., Mott R., Hurst L.D., Harberd N.P. (2014). Environmentally responsive genome-wide accumulation of de novo Arabidopsis thaliana mutations and epimutations. Genome Res..

[153] Wang W.S., Pan Y.J., Zhao X.Q., Dwivedi D., Zhu L.H., Ali J., Fu B.Y., Li Z.K. (2011). Drought-induced site-specific DNA methylation and its association with drought tolerance in rice (Oryza sativa L.). J. Exp. Bot..

[154] Paun O., Bateman R.M., Fay M.F., Hedren M., Civeyrel L., Chase M.W. (2010). Stable epigenetic effects impact adaptation in allopolyploid orchids (Dactylorhiza: Orchidaceae). Mol. Biol. Evol..

[155] Hofmeister B.T., Lee K., Rohr N.A., Hall D.W., Schmitz R.J. (2017). Stable inheritance of DNA methylation allows creation of epigenotype maps and the study of epiallele inheritance patterns in the absence of genetic variation. Genome Biol..

[156] Vasquez-Robinet C., Mane S.P., Ulanov A.V., Watkinson J.I., Stromberg V.K., De Koeyer D., Schafleitner R., Willmot D.B., Bonierbale M., Bohnert H.J. (2008). Physiological and molecular adaptations to drought in Andean potato genotypes. J. Exp. Bot..

[157] Lacal I., Ventura R. (2018). Epigenetic Inheritance: Concepts, Mechanisms and Perspectives. Front Mol Neurosci..

[158] Norouzitallab P., Baruah K., Vanrompay D., Bossier P. (2019). Can epigenetics translate environmental cues into phenotypes?. Sci. Total Environ..

[159] Reynolds M., Langridge P. (2016). Physiological breeding. Curr. Opin. Plant Biol..

[160] Fiaz S., Ahmad S., Noor M.A., Wang X., Younas A., Riaz A., Riaz A., Ali F. (2019). Applications of the CRISPR/Cas9 System for Rice Grain Quality Improvement: Perspectives and Opportunities. Int. J. Mol. Sci..

[161] Sheng Z., Fiaz S., Li Q., Chen W., Wei X., Xie L., Jiao G., Shao G., Tang S., Wang J. (2019). Molecular breeding of fragrant early-season hybrid rice using the BADH2 gene. Pak. J. Bot..

[162] Barman H.N., Sheng Z., Fiaz S., Zhong M., Wu Y., Cai Y., Wang W., Jiao G., Tang S., Wei X. (2019). Generation of a new thermo-sensitive genic male sterile rice line by targeted mutagenesis of TMS5 gene through CRISPR/Cas9 system. BMC Plant Biol..

[163] Qi L.S., Larson M.H., Gilbert L.A., Doudna J.A., Weissman J.S., Arkin A.P., Lim W.A. (2013). Repurposing CRISPR as an RNA-guided platform for sequence-specific control of gene expression. Cell.

[164] Gilbert L.A., Larson M.H., Morsut L., Liu Z., Brar G.A., Torres S.E., Stern-Ginossar N., Brandman O., Whitehead E.H., Doudna J.A. (2013). CRISPR-Mediated Modular RNA-Guided Regulation of Transcription in Eukaryotes. Cell.

[165] Piatek A., Ali Z., Baazim H., Li L., Abulfaraj A., Al-Shareef S., Aouida M., Mahfouz M.M. (2015). RNA-guided transcriptional regulation in planta via synthetic dCas9-based transcription factors. Plant Biotechnol. J..

[166] Duan Y.-B., Li J., Qin R.-Y., Xu R.-F., Li H., Yang Y.-C., Ma H., Li L., Wei P.-C., Yang J.-B. (2016). Identification of a regulatory element responsible for salt induction of rice OsRAV2 through ex situ and in situ promoter analysis. Plant Mol. Biol..

[167] Lou D., Wang H., Liang G., Yu D. (2017). OsSAPK2 Confers Abscisic Acid Sensitivity and Tolerance to Drought Stress in Rice. Front. Plant Sci..

[168] Fiaz S., Wang X., Younas A., Alharthi B., Riaz A., Ali H. (2020). Apomixis and strategies to induce apomixis to preserve hybrid vigor for multiple generations. Gm Crop. Food.

[169] Nguyen D., Rieu I., Mariani C., van Dam N.M. (2016). How plants handle multiple stresses: Hormonal interactions underlying responses to abiotic stress and insect herbivory. Plant Mol. Biol..

[170] Reinders J., Wulff B.B., Mirouze M., Mari-Ordonez A., Dapp M., Rozhon W., Bucher E., Theiler G., Paszkowski J. (2009). Compromised stability of DNA methylation and transposon immobilization in mosaic Arabidopsis epigenomes. Genes Dev..

[171] Johannes F., Porcher E., Teixeira F.K., Saliba-Colombani V., Simon M., Agier N., Bulski A., Albuisson J., Heredia F., Audigier P. (2009). Assessing the impact of transgenerational epigenetic variation on complex traits. PLoS Genet..

[172] Yamauchi T., Johzuka-Hisatomi Y., Terada R., Nakamura I., Iida S. (2014). The MET1b gene encoding a maintenance DNA methyltransferase is indispensable for normal development in rice. Plant Mol. Biol..

[173] Hung Y.-H., Liu F., Zhang X.-Q., Xiao W., Hsieh T.-F. (2018). Sexual and Non-sexual Reproduction: Inheritance and Stability of Epigenetic Variations and Consequences for Breeding Application. Adv. Bot. Res..

[174] Danchin E., Pocheville A., Rey O., Pujol B., Blanchet S. (2019). Epigenetically facilitated mutational assimilation: Epigenetics as a hub within the inclusive evolutionary synthesis. Biol. Rev..

[175] Rendina González A.P., Preite V., Verhoeven K.J.F., Latzel V. (2018). Transgenerational Effects and Epigenetic Memory in the Clonal Plant Trifolium repens. Front. Plant Sci..

[176] Xu J., Tanino K.K., Robinson S.J. (2016). Stable Epigenetic Variants Selected from an Induced Hypomethylated Fragaria vesca Population. Front. Plant Sci..

[177] Gourcilleau D., Mousset M., Latutrie M., Marin S., Delaunay A., Maury S., Pujol B. (2019). Assessing Global DNA Methylation Changes Associated with Plasticity in Seven Highly Inbred Lines of Snapdragon Plants (Antirrhinum majus). Genes.

